# The Development of Statewide Policies and Procedures to Implement Telehealth for Part C Service Delivery

**DOI:** 10.5195/ijt.2016.6206

**Published:** 2016-12-15

**Authors:** BETH COLE, ARLENE STREDLER-BROWN, BECKI COHILL, KRISTINA BLAISER, DIANE BEHL, SHARON RINGWALT

**Affiliations:** 1 EARLY INTERVENTION COLORADO STATE STAFF, COLORADO, USA; 2 DEPARTMENT OF SPEECH, LANGUAGE, AND HEARING SCIENCES, UNIVERSITY OF COLORADO, BOULDER, COLORADO, USA; 3 DEPARTMENT OF OCCUPATIONAL THERAPY, UNIVERSITY OF ST. AUGUSTINE, SAN MARCOS, CALIFORNIA, USA; 4 DEPARTMENT OF COMMUNICATION SCIENCES AND DISORDERS, IDAHO STATE UNIVERSITY, MERIDIAN, IDAHO, USA; 5 NATIONAL CENTER FOR HEARING ASSESSMENT AND MANAGEMENT, UTAH STATE UNIVERSITY, LOGAN, UTAH, USA; 6 EARLY CHILDHOOD TECHNICAL ASSISTANCE CENTER, CHAPEL HILL, NORTH CAROLINA, USA

**Keywords:** Early intervention (EI), Individuals with Disabilities Education Act (IDEA), Telehealth

## Abstract

The use of telehealth has been discussed nationally as an option to address provider shortages for children, birth through two, enrolled in Part C of the Individuals with Disabilities Education Act (IDEA) Early Intervention (EI) programs. Telehealth is an evidence-based service delivery model which can be used to remove barriers in providing EI services to children and their families. In 2016, Colorado’s Part C Early Intervention (EI) program began allowing the use of telehealth as an option for providers to conduct sessions with children and their caregivers. This article outlines the process taken to develop the necessary requirements and supports for telehealth to be incorporated into EI current practice.

Telehealth^1^ or the provision of services via videoconferencing technology, has become increasingly popular as a service delivery option for early intervention (EI), and supports the EI model in many ways. The use of telehealth as a service delivery model reinforces parents’ self-efficacy. According to Meyer et al. (2002), “Parents know that they are in control of their child’s services [and they are] viewed as being integral to successful intervention” (p. 415). Telehealth not only facilitates the therapists’ task of coaching families, recent studies have shown that coaching behaviors are actually enhanced in telehealth sessions because the provider is not physically present in the home (Blaiser, Behl, Callow-Heusser, & White, 2013). This is important to note because, while coaching empowers family members to know how to support their child’s development throughout their everyday routines, many early intervention providers do not actually implement coaching in their everyday practice (Campbell et al., 2009; Woods & Friedman, 2012).

According to Clark (2010), “The more information that we have flowing, the faster we can respond to change and the more relevant we can remain to everyone” (p. 854). Imagine problem solving a difficult bedtime routine with a family through the use of telehealth, offering suggestions during the actual routine, without having to be physically present in the family’s house during bedtime. Using telehealth as a service delivery model expands the ability of a provider to meet the needs of children and their families through the use of technology.

Telehealth can be used in early intervention in a wide variety of situations to:

- hold sessions during inclement weather;
- account for provider shortages;
- conduct sessions when the therapist is ill, yet able to work, and the child has a compromised immune system;
- bring another parent or caregiver into the session using three computers in three sites;
- bring a specialist (e.g., a mental health or vision specialist) into a session;
- offer consultations with providers who have specialized expertise (e.g., autism, feeding, assistive technology) when that expertise may not be available locally;
- supervise and coach an in-person provider;
- ease transition into a new foster care or adoption setting; and
  continue visits during vacations in accord with state and federal laws, regulations, and statutes and licensure board(s) requirements (in the state where the provider is located and where the family is located, if in a different state).

Through Part C of the Individuals with Disabilities Education Act (IDEA), early intervention (EI) programs serve families with children from birth through two years of age with disabilities and developmental delays. The demand for early intervention in Colorado continues to increase; however, personnel shortages, especially in the rural areas, limit the number of children who are able to access appropriate services (Dashboard Report for Indicator 1, 2011). These provider shortages mirror what is seen nationally (Cason, Behl, & Ringwalt, 2012; A.J. Pappanikou Center for Excellence in Developmental Disabilities, 2004), and impacts the services that are available for children and families enrolled in Part C EI. In Colorado, the shortages appear in all disciplines, but are especially acute for specialists in the areas of mental health, low vision, assistive technology, and hearing. Telehealth has become increasingly popular as a service delivery option for early intervention. Heimerl and Rasch (2009) reported that for children who live in rural areas or communities experiencing a shortage of pediatric-trained occupational therapists, telehealth is a feasible option to provide access to services. Increasing the availability of telehealth in Colorado, the legislature passed a bill in 2015 requiring insurance companies to pay for services provided via telehealth for all communities including those with a population of 100,000 or more (CRS 10-16-123).

Although barriers exist, many states are incorporating telehealth into their early intervention IDEA Part C services to improve access to services and overcome personnel shortages. Policy development, education of stakeholders, research, utilization of secure and private delivery platforms and advocacy may facilitate more widespread adoption of telehealth (Cason, Behl, & Ringwalt, 2012). In 2013, Colorado began exploring the use of telehealth to address the issue of provider shortages. Colorado State Part C partners launched an initiative to develop comprehensive policies and procedures for the statewide implementation of telehealth. A primary driver for policy development was to ensure that providers are well trained and deliver services in an effective and safe manner.

## METHODS

The Colorado initiative for telehealth implementation for the Part C Early Intervention program followed a disciplined protocol. Stakeholders and their role on the task force are outlined in Table 1.

### COLORADO TELEHEALTH IMPLEMENTATION PROTOCOL

1. **Establishment of a Task Force.** The Telehealth Policies and Procedures Task Force was assembled to facilitate the Colorado’s Early Intervention program use of telehealth. It was deemed important that those participating on the Task Force believed in the use of telehealth as a service method for EI, and that there was broad representation of stakeholders that leveraged statewide and national telehealth expertise.
2. **Policy Review and Creation of Policies and Procedures.** The Task Force members began by reviewing the Colorado Rules for early intervention (12 CCR 2509-10) to determine what needed to be added and/or modified to accommodate telehealth. Proposed changes needed to be identified in sufficient time for public comment prior to submission to the Office of Special Education Programs (OSEP) for approval. The Task Force members also reviewed telehealth billing information (allowed for speech therapy) and best practices for service provision. Since Colorado Medicaid pays an additional amount for telehealth sessions, the Colorado EI program decided to allow providers to bill an additional $10 when a session is conducted via telehealth. This charge is intended to help defray some of the costs of the technology, such as a HIPAA-compliant web-based platform.
3. **Piloting telehealth.** Colorado selected its local program in Pueblo to be a pilot site. This program was selected for several reasons. It serves only one geographically diverse county comprised of rural (plains), mountain, and urban settings. In addition, the entire county has relatively reliable broadband available. An experienced staff, led by a director who with enthusiasm for telehealth, serves an average of 173 children through its EI program. Additionally, one of the providers serving this county was completing a doctoral degree; the provider’s dissertation focused on the use of telehealth for occupational therapy in early intervention. Pilot site participants included early intervention providers from a variety of disciplines: occupational therapy, physical therapy, education of the deaf and hard of hearing, social-emotional, and speech-language pathology, in addition to service coordinators and administration.
4. **Development of Training for EI providers and administrators.** A review of available resources pertaining to telehealth for EI revealed no published training materials specific to this population. However, personal communications with colleagues in the EI field revealed that the National Center for Hearing Assessment and Management (NCHAM) at Utah State University had developed an online resource guide for telehealth, and was in the process of developing online training courses for administrators, direct service providers, and families. Associated Colorado EI providers were invited to be part of the Colorado-based field testing of the training content which was tested via an in-person, two-day training session. The training included video examples of telehealth, small group discussion activities, and didactic instruction. Content covered the role of telehealth in supporting natural environments, technology hardware and software, privacy and security, and state and federal licensure requirements. In all, twenty Colorado EI providers and administrators from across the state attended the two-day training in Pueblo, Colorado.

## RESULTS

The multi-step process that comprised the state initiative yielded the following outcomes.

### OUTCOMES OF THE INITIAL TRAINING

**Figure 1:**
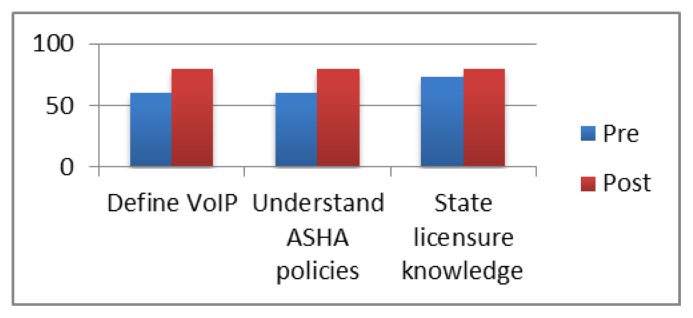
Percentage of providers who were able to correctly respond to telehealth quiz questions before and after the in-service training.

**Figure 2:**
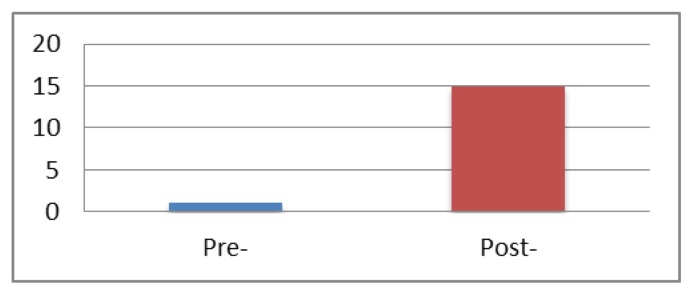
Number of providers who were able to identify four privacy and security factors to consider before and after the in-service training.

Pre- and post- assessments were administered to evaluate the outcomes of the onsite training. An internet-based survey was sent to all participants before and after the two-day training. Fifteen attendees completed the pre-training survey and 10 attendees completed the post-training survey. Survey questions focused on the participants’ knowledge of issues pertinent to the implementation of telehealth (e.g., privacy, internet protocols and coaching techniques). Figures 1 and 2 summarize the results from this pre-post training survey.

The information gained from the onsite training was applied to the creation of three free online training courses developed by NCHAM, available at http://TI101.org (Blaiser, Behl, Edwards, Olsen & Cook, 2015). The course for administrators includes content on privacy and security, costs and selection of software and hardware, and strategies for supporting EI providers. The course for direct service providers includes sample video recordings of telehealth sessions and sample lesson plans, content on how to incorporate coaching and natural environments into telehealth, and strategies to troubleshoot technology problems. The course designed for families explains how telehealth sessions work, the family’s role in telehealth, and potential benefits and challenges.

### PILOT SITE OUTCOMES

The purpose of the pilot initiative was for providers in the Part C agency to use telehealth to provide services to children in early intervention as well as to discover the issues and challenges that the Task Force would need to consider. One significant challenge was the reluctance of case managers (Part C service coordinators) and families to agree to use telehealth. Much of their reluctance stemmed from a lack of understanding of the process and a paucity of evidence supporting the use of telehealth. In response to this hesitancy, the Task Force members developed a brochure explaining telehealth, with a change in terminology. After much discussion, the Task Force agreed that the term “Live Video Visits” would be more descriptive of a telehealth session. The brochure was given to prospective families, as well as to all Service Coordinators. The pilot site reported that the brochure facilitated an increase in service coordinators’ willingness to talk to families about telehealth. Three families used telehealth for services in the pilot study, and all were very satisfied with the services.

As a result of this success, the term ‘Live Video Visits’ will be used in all Colorado public awareness materials. However, while the brochure helped some families to try telehealth, overcoming family members’ reluctance continues to be a challenge.

### OUTCOMES FROM TASK FORCE MEETINGS

The initial Task Force meetings indicated that very few changes needed to be made in the State Part C Early Intervention Rules (12 CCR 2509-10) to allow local programs to utilize telehealth as a method for serving children, birth through two years of age, and their family members. Telehealth was added as a service delivery method and not as a separate service. Telehealth could be employed by any qualified provider, once they met the requirements to utilize telehealth. The major change made to the Rules was to require any provider who planned to bill for telehealth to initially complete state-sponsored training. These changes went into effect July 1, 2016.

The Task Force also developed a consent form as a formalized assurance that families understood what telehealth meant and knew what to expect. The Task Force decided that a second consent form was necessary to gain the permission from a parent or guardian to share a child’s session recordings with other caregivers. Likewise, family members would need to obtain their provider’s consent if they wanted to share a recording with any other individuals.

The Task Force created a checklist for local programs to use to ensure that providers of telehealth working in early intervention were adequately prepared. The checklist included 18 items representing five issues: (a) therapist training; (b) appropriateness of telehealth for a child and his/her family; (c) security; (d) the environment; and (e) a contingency plan if a session did not have an adequate internet connection. These issues align with recommendations from Cason (2011) that stated, “Extensive training materials for caregivers and providers should be provided, in addition ongoing training and competency standards should be in place to ensure that providers demonstrate technical and therapeutic skills necessary for the delivery of EI services using telehealth” (p. 26).

## DISCUSSION

As part of the effort to roll out telehealth statewide, Colorado has had the opportunity to modify actions and create documents that were determined to be necessary and useful. This section discusses the major lessons the Task Force learned and what occurred in response to those lessons.

### TRAINING

#### MODULES

The Colorado Task Force determined that there was a need to develop their own training in telehealth to highlight the state’s philosophy about the benefits of telehealth and to incorporate state-specific policies and practices. The resultant four modules developed by the Task Force focused on: (a) establishing that telehealth is just a different way of providing services that has many advantages for both families and providers; (b) the fundamentals of telehealth and EI-related legislation, billing, required forms, etc.; (c) details on how to plan for and conduct a telehealth session; and (d) reviewing actual telehealth sessions (featuring Task Force members) to better understand how to conduct a successful session.

#### TECHNOLOGY

The training discuss telehealth technology and ways to evaluate the best option for each provider. The training also discusses use of hot spots and the ability to boost bandwidth to ensure a better connection. Two of the families who were being seen remotely during the pilot used hot spots and the sessions had no interruptions. In addition, the training includes various procedures a provider can use to trouble-shoot a connection, including a contingency plan when the connection is not working well. The last issue discusses recordings – who makes them, how they may be used, and how they must be stored.

#### PRIVACY AND SECURITY

Training materials will clearly outline that providers are required to use a HIPAA-compliant platform for sessions. Due to the fact that platforms are constantly changing, the state program is refraining from making recommendations. The training will identify some of the most common software programs that are known to be HIPAA compliant, with the caveat that the available platforms are changing constantly and those identified are only some of the possible options that may be available. Finally, the early intervention program is working with a statewide agency that has an assistive technology loan bank so that technology, such as hot spots and iPads will be available to support the use of telehealth in Colorado.

#### DISPELLING MYTHS

The online training offered to Colorado practitioners contains a section on dispelling myths about telehealth. One myth is that telehealth is “lesser than” in-person services. Research is demonstrating that this is not true, and that in actuality, children’s outcomes are sometimes even better than those for children who are receiving services by an in-person therapist (Blaiser & Behl, 2015; Baharav & Reiser, 2010). Another myth is that providers aren’t able to build rapport with families when telehealth is used. When one looks at the relationships developed over social media, this myth can be dispelled. Finally, there is a myth that technology is very expensive and difficult to use. There are so many different platforms that meet the needs of providers who are utilizing telehealth that finding one that meets a therapist’s and family’s needs is increasingly easier and less expensive.

#### COACHING

While coaching is a necessary skill for providing services via telehealth, the Task Force decided to not require specific training on this topic. This was due to the fact that coaching is considered best practice in early intervention and all therapists should be using it, regardless of whether they are in the home or conducting a remote session.

The online training will be housed on a platform managed by the EI state staff. Updates will be managed in-house and available for free to providers in Colorado or in other states.

## NEXT STEPS

Once the use of telehealth becomes a service delivery method that providers can utilize to serve children in Colorado’s EI program, the Task Force will begin to collect data to determine how the use of telehealth impacts EI services. The hope is that children in early intervention will receive the frequency and quality of services that is closer to what is indicated on the child’s Individualized Family Service Plan (IFSP), due to increased access to providers and fewer missed visits. The Task Force also wants to learn if some families choose to use telehealth more frequently than in-person visits (as was the case in the pilot study), and the ways in which provider and family attitudes toward telehealth evolve. The Task Force will be responsive to any data that suggest the need for changes. State EI staff will also participate in statewide efforts to bring broadband to all areas of the state, as internet connectivity is anticipated to continue to be one of the largest barriers to the use of telehealth in Colorado’s EI program.

## Figures and Tables

**Table 1:** Stakeholders and Role on Task Force

Representative	Role on Task Force
State Early Intervention Staff	Shared background on State policies and procedures Modified Colorado Rules for EI, (12 CCR 2509-10) Developed required forms for local programs and providers
Community Early Intervention Providers (Occupational Therapists, Physical Therapists, Speech-Language Pathologists)	Reported on experience utilizing telehealth Provided input into forms Developed public awareness materials Provided input on provider training
Representatives from the Community Pilot Project	Reported success of telehealth Reported challenges using telehealth Offered suggestions regarding public awareness materials and forms
Researchers	Conducted literature review Identified national initiatives supporting telehealth
Administrative and Local Programs	Provided input regarding impact on local programs
Local Program IT Director and HIPAA Officer	Provided information about technology Offered information about HIPAA and FERPA (Federal Educational Rights and Privacy Act) privacy considerations

## Footnotes

1 There are many terms used for telehealth – teleintervention, telemedicine, etc. Due to insurance legislation in Colorado that uses the term “telehealth”, that is the term Colorado adopted and the term that will be used throughout this article.
